# Employment and Compliance with Pandemic Influenza Mitigation Recommendations

**DOI:** 10.3201/eid1602.090638

**Published:** 2010-02

**Authors:** Kelly D. Blake, Robert J. Blendon, Kasisomayajula Viswanath

**Affiliations:** Harvard School of Public Health, Boston, Massachusetts, USA (K.D. Blake, R.J. Blendon, K. Viswanath); Dana-Farber Cancer Institute, Boston (K.D. Blake, K. Viswanath)

**Keywords:** Pandemic, preparedness, employment, workplace, planning, mitigation, influenza, expedite, research

## Abstract

In the event of a serious pandemic influenza outbreak, businesses must play a key role in protecting employees' health and safety. With regard to pandemic influenza mitigation recommendations requiring social distancing, we examined whether some US employees would disproportionately fail to comply because of job insecurity and financial problems associated with missing work. We used the 2006 Harvard School of Public Health Pandemic Influenza Survey and multivariable logistic regression to determine whether employment characteristics such as inability to work from home, lack of pay when absent from work, and self-employment would be associated with less ability to comply with recommendations. We found that inability to work from home, lack of paid sick leave, and income are associated with working adults’ ability to comply and should be major targets for workplace interventions in the event of a serious outbreak.

## Abstract 2

Noncompliance may result from job insecurity and financial problems associated with missing work.

The world needs a detailed operational blueprint for the best way to get through 12–24 months of a pandemic influenza outbreak; that type of planning must be on the agenda of every public health agency, school board, state legislature, and business (*1*). In January 2008, the Centers for Disease Control and Prevention outlined several recommendations (*2*). In the event of a serious outbreak, employers must play a key role in protecting employees’ health and safety (*2*). Specifically, businesses should “forecast and allow for employee absences during a pandemic due to factors such as personal illness, family member illness, community containment measures and quarantines, school and/or business closures, and public transportation closures” and workers should “plan for the possible reduction or loss of income if [they are] unable to work or if place of employment is closed” (*2*).

Should social distancing from the workplace become a reality, some members of the US workforce may be disproportionately vulnerable to compliance failure and negative outcomes of an influenza pandemic because of real and perceived job insecurity and financial problems associated with missing work. Previous research suggests that compliance with recommendations in emergency situations reflects the interaction of many modifiable and nonmodifiable factors, including how persons perceive their personal and family risk, what resources they have available, what negative consequences they anticipate as a result of compliance, their socioeconomic status, and how well official planning efforts are organized (*3*–*6*). Attitudes toward the use of social distancing to mitigate outbreaks of severe acute respiratory syndrome, smallpox, or avian influenza may be influenced by largely modifiable problems that people associate with isolation, such as not being able to get healthcare or prescription drugs and losing pay or jobs for missing work (*7*). Indeed, more than one third of US employees say that they would not get paid if they had to stay home from work because of a severe outbreak of pandemic influenza, and less than one third believe that they could work from home for 1 month (*8*).

We hypothesized that working adults who are unable to work from home and who do not have sick leave will have less ability to comply with pandemic influenza isolation recommendations that require missing work because of fear of losing their job or business and serious financial problems that would arise from missing work. To test our hypothesis, we assessed the relative independent contribution of selected employment and sociodemographic characteristics on working adults’ ability to comply with pandemic influenza mitigation strategies involving workplace isolation.

## Methods

### Data

We used data from the 2006 Harvard School of Public Health (HSPH) Pandemic Influenza Survey, a random digit–dial survey sponsored by the HSPH Project on the Public and Biological Security. The survey was conducted to provide information with regard to the public’s reaction to the possible use of social distancing and other nonpharmaceutical interventions during a severe outbreak of pandemic influenza. Survey questions assessed willingness and ability to comply with public health recommendations in 3 domains: home, school, and work.

The target population was adults >18 years of age, who lived in the United States. During September 28 through October 25, 2006, International Communications Research (Media, PA, USA) surveyed a representative sample of adults >18 years of age, including an oversample of adults with children <18 years of age in the household. Sampling procedures are described elsewhere (*8*). Response rate was 36% and cooperation rate was 75%, which produced a total of 1,697 completed interviews (*8*). Because adults with children were oversampled to gauge the possible effect of community mitigation on families, data were weighted to reflect the actual proportion of the total adult population. In addition, to compensate for nonresponse bias and unequal probability of selection and to ensure that demographic groups were represented in their actual proportion in the adult population, we weighted sample data to the most recent US census data available from the Current Population Survey (www.census.gov/cps) for gender, age, race, region, and education.

Employment characteristics for the full sample were 50% employed full time, 13% employed part time, 36% unemployed, and 1% unknown. Our study focused on employment-related constraints that may limit ability to comply with isolation recommendations; therefore, we analyzed responses from only the 1,101 respondents who were employed either full or part time (Table 1). A small percentage of employed respondents refused to answer some items in the survey (e.g., 15% refused income, 3% education, 2% race).

**Table 1:** Sample characteristics of 1,101 employed respondents, 2006 Harvard School of Public Health Pandemic Influenza Survey*

Characteristic	% Respondents
Female	54
Age, y	
18–30	28
31–50	48
>51	24
Annual household Income	
<$30,000	18
$30,000–$49,000	19
$50,000–$74,000	19
>$75,000	29
Education	
Less than HS	12
HS graduate or HS plus technical school	29
Some college, no degree	25
College degree or more	31
Race/ethnicity	
African-American	11
Hispanic	14
Other	7
White	66
Residence	
Urban	73
Rural	27
Employment	
Part time	21
Full time	79

*HS, high school. Alll samples are weighted. Entries may not total 100% because of refused or missing responses.

### Outcome Variables: Indicators of Ability to Comply

Employed respondents were asked a series of questions to assess real or perceived constraints with regard to their ability to comply with pandemic influenza mitigation strategies that require isolation from the workplace. The 4 dependent variables were positive responses to the following statements: “It is likely that I or a member of my household would lose a job or business as a result of having to stay home for 7–10 days” (dichotomized; “very” and “somewhat” likely represented a positive response and “not too” and “not at all” likely represented a negative response); and “I would have serious financial problems if I stayed away from work for 7–10 days, 1 month, or 3 months.” The latter questions used a split sample, whereby only respondents who answered “no” to the 7–10 day duration were asked about financial problems that they would have at 1 month, and only those who answered “no” to having serious financial problems at 1 month were asked about the 3-month period. Many unadjusted prevalence estimates for each outcome variable (Table 2) have been described in a descriptive study that used the 2006 HSPH Pandemic Influenza Survey (*8*).

**Table 2:** Prevalence estimates for responses of 1,101 employed respondents (unadjusted), 2006 Harvard School of Public Health Pandemic Influenza Survey*

Variable	No. responses	% Yes	% No	% Don’t know
Outcome variables representing ability to comply with pandemic influenza mitigation recommendations				
If you were asked to stay home for 7–10 days and avoid contact with anyone outside your household, would you or someone in your household lose your job or business?	1,073	28	71	1
Would it become a serious financial problem if you stayed out of work for 7–10 days?	1,072	25	74	1
Would it become a serious financial problem if you stayed out of work for 1 month?	806†	42	55	2
Would it become a serious financial problem if you stayed out of work for 3 months?	464‡	45	51	4
Key predictor variables representing employment-related constraints on compliance				
Unable to work from home for 1 month in the event of a serious outbreak	1,073	69	29	2
Would not be paid if kept from work because of a serious outbreak	1,071	42	35	22
Self-employed	1,072	16	84	–

*All estimates are weighted. Cell counts may not total 100% due to refused or missing responses. Sample size for each question varies due to refused and missing responses. †Split sample; question asked only of those who responded “no” or “don’t know” to financial problems after 7–10 days. ‡Split sample; question asked only of those who responded “no” or “don’t know” to financial problems after 1 month.

### Predictor Variables

To assess the effect of potentially modifiable employment-related constraints on compliance with recommendations that require missing work, we chose key predictor variables that represented selected employment characteristics; i.e., inability to work from home, lack of pay when absent from work, and self-employment status (Table 2). With regard to employment-related constraints, <1% of respondents refused to answer 2 questions.

### Covariates

To assess the potentially disproportionate difficulties that low-income and urban populations may face if asked to stay home from work in the event of a serious outbreak, we included income and urban residence in all models. Other sociodemographic and personal characteristics assessed were education, race/ethnicity, age, sex, self-reported health status, and self-reported knowledge of pandemic influenza.

### Statistical Analysis

Structured analytical approaches have improved predictions of behavior in emergency situations, modeling the joint effects of several factors on planned behavior (*3*). Therefore, to identify factors that may lead to disproportionate vulnerability in the event of a serious outbreak, we used multivariable logistic regression to model the predicted probability that some groups of working adults (delineated by employment characteristics such as inability to work from home, lack of pay when absent from work, and self-employment) may be less able than identified referent groups to comply with pandemic influenza mitigation strategies that require voluntary isolation from work.

Each outcome used 1 full model; all models controlled simultaneously for all key predictors as well as covariates such as income, urban residence, age, race/ethnicity, education, gender, self-reported health status, and self-reported knowledge of pandemic influenza. We conducted a complete case analysis and analyzed only those 1,101 respondents who reported being employed either full or part time. Tests of significance were estimated at p<0.05, and 95% confidence intervals (CIs) were reported for all odds ratios (ORs). To adjust for unequal probabilities of selection and for potential nonresponse bias, we applied individual weighting factors to all estimates. The analysis was conducted with SAS software version 9.2 (Cary, NC, USA) by using the PROC SURVEYLOGISTIC procedure and probability sampling weights.

## Results

### Perceived Likelihood of Losing Job or Business

Of the employed respondents, 28% reported that they likely would lose their job or business as a result of having to stay home from work for 7–10 days in the event of a pandemic influenza outbreak (Table 2). Our multivariable models elucidated differential vulnerability by lack of paid sick leave, income level of respondents, and urban residence (Table 3). Those respondents who would not be paid if kept from work were almost 5× more likely than those who would receive pay (OR 4.72) to say that they would likely lose their job or business as a result of having to stay home from work for 7–10 days.

**Table 3:** Likelihood of compliance with work-related pandemic influenza isolation strategies, by employment-related constraints and sociodemographics*

Variable	Referent	Likelihood,† OR (95% CI)			
		Lose job or business, n = 928‡	Serious financial problems		
			7–10 d, n = 927§	1 mo, n = 754¶	3 mo, n = 472#
Employment-related constraints					
Unable to work from home for 1 mo	Able	0.99 (0.63–1.56)	1.57 (1.02–2.51)**	**1.91 (1.30–2.79)††**	1.65 (1.06–2.52)**
Would not be paid if kept from work	Paid	**4.72 (2.94–7.57)††**	**3.23 (2.03–5.13)††**	**2.93 (2.07–4.14)††**	**1.75 (1.14–2.62)‡‡**
Self-employed	Works for someone else	1.09 (0.64–1.85)	**2.09 (1.25–3.49)‡‡**	0.68 (0.42–1.14)	0.66 (0.38–1.16)
Sociodemographic characteristics					
Income	>$75,000				
<$30,000		**4.31 (2.43–7.63)††**	**3.26 (1.85–5.75)††**	**3.29 (1.78–6.05)††**	**3.52 (1.38–8.98)‡‡**
$30,000–$49,000		1.70 (1.01–3.02)**	1.57 (0.93–2.64)	**2.93 (1.81–4.75)††**	1.46 (0.79–2.69)
$50,000–$74,000		**2.08 (1.25–3.48)‡‡**	1.09 (0.64–1.84)	**1.89 (1.25–2.88)‡‡**	1.27 (0.78–2.07)
Urban residence	Rural	1.66 (1.07–2.56)**	1.30 (0.84–2.01)	1.14 (0.77–1.68)	0.66 (0.41–1.06)
Education	College				
Less than HS		2.40 (0.84–6.80)	1.73 (0.62–4.80)	1.24 (0.36–4.28)	0.45 (0.06–3.29)
HS graduate or HS plus technical school		**2.03 (1.17–3.51)‡‡**	1.62 (0.95–2.75)	1.17 (0.69–1.99)	0.41 (0.20–1.07)
Some college		1.15 (0.63–2.05)	1.25 (0.73–2.14)	0.89 (0.55–1.42)	0.71 (0.38–1.31)
Race/Ethnicity	White				
African-American		1.74 (0.92–3.29)	0.56 (0.26–1.18)	1.51 (0.77–2.95)	0.73 (0.31–1.70)
Hispanic		1.55 (0.83–2.88)	0.65 (0.32–1.34)	0.74 (0.37–1.48)	1.52 (0.65–3.57)
Other		2.23 (0.92–5.43)	1.22 (0.45–3.26)	0.71 (0.27–1.86)	0.81 (0.29–2.31)
Age, y	>51				
18–30		1.99 (1.09–3.66)**	1.08 (0.56–2.05)	0.73 (0.40–1.32)	1.54 (0.75–3.15)
31–50		1.09 (0.71–1.71)	1.49 (0.96–2.33)	1.04 (0.71–1.53)	1.38 (0.88–2.16)
Gender	F	0.77 (0.53–1.13)	0.84 (0.57–1.22)	1.07 (0.76–1.51)	1.07 (0.72–1.61)
Good health status	Poor health	0.58 (0.29–1.13)	0.50 (0.25–0.97)**	0.96 (0.40–2.29)	0.60 (0.22–1.69)
Knowledge of pandemic influenza	Never heard of	0.59 (0.37–0.96)	0.79 (0.49–1.30)	1.23 (0.73–2.06)	1.02 (0.53–1.94)
–2LL		735.72	740.38	860.05	605.98

*Multivariable fitted logistic regression models describing the odds that some groups may be less able than identified referent groups to comply with pandemic influenza mitigation strategies that require voluntary isolation from work. All estimates are weighted and controlled for age, race/ethnicity, education, gender, self-reported health status, and self-reported knowledge of pandemic influenza. OR, odds ratio; CI, confidence interval; HS, high school; LL, log likelihood. **Boldface** indicates significance at p<0.05. †Response of persons employed full or part time to “If pandemic influenza remained in your community for some time, health officials might recommend that people stay home from work so they do not catch or spread the disease.” ‡I or a member of my household would lose job or business as a result of having to stay home for 7–10 days. §I or a member of my household would have serious financial problems if I stayed away from work for the following period of time. ¶Split sample; question asked only of those who responded “no” or “don’t know” to financial problems after 7–10 days. #Split sample; question asked only of those who responded “no” or “don’t know” to financial problems after 1 month. **p<0.05. ††p<0.0001. ‡‡p<0.01.

Respondent income also was associated with reported likelihood of losing a job or business. Those who earned <$30,000 per year were 4× more likely than those who earned >$75,000 per year (OR 4.31) and those who earned $30,000–$49,000 per year and $50,000–$74,000 per year were ≈2× more likely than those who earned >$75,000 per year (ORs 1.70 and 2.08, respectively) to say that they would likely lose their job or business as a result of having to miss work in the event of a serious outbreak.

Urban residence was associated with limited ability to comply with recommendations that require missing work. Respondents living in urban areas were ≈70% more likely than those living in rural areas to say that they would likely lose their job or business as a result of having to stay home for 7–10 days in the event of an outbreak (OR 1.66).

### Perceived Likelihood of Experiencing Serious Financial Problems

Certain employment characteristics and respondent income levels were associated with the likelihood that working adults would experience serious financial problems and thus be less able to comply with isolation recommendations, if required to miss work for long periods of time (Table 3). Respondents who were self-employed were twice as likely as those who worked for an employer to say that they would experience serious financial difficulties if isolated from work for 7–10 days (OR 2.09). Those who were not able to work from home were significantly more likely than those who were able to work from home to say that they would experience serious financial problems if isolated from work for durations of 7–10 days, 1 month, and 3 months (ORs 1.57, 1.91, 1.65, respectively).

Respondent income also was associated with likelihood of experiencing serious financial problems if the respondent were kept from work because of an outbreak of pandemic influenza, although the models showed interesting patterns, depending on the duration of isolation. If isolated from work for 7–10 days, those who earned <$30,000 per year were 3× more likely than those who earned >$75,000 per year to say that they would experience substantial financial problems (OR 3.26). At 1 month of isolation, all those in low- and middle-income groups were significantly more likely than those in the highest income group to say that they would experience serious financial problems if kept from work. There was a fine income gradient; those earning <$30,000 per year were 3.29× more likely, those earning $30,000–$49,000 per year were 2.93× more likely, and those earning $50,000–$74,000 per year were 1.89× more likely than those earning >$75,000 per year to say that staying away from work for 1 month would pose serious financial problems. At 3 months of isolation, the trend shifted somewhat. Low-income workers were still significantly more likely than high-income workers to say that they would have serious financial problems if isolated from work (OR 3.52), indicating disproportionate vulnerability for low-income populations across all durations of isolation. However, at 3 months, middle-income workers would not be more or less likely than those earning >$75,000 to say that they would experience serious financial problems, indicating that a 3-month period of isolation would likely be difficult for those in all income groups, including those in the highest income categories.

## Discussion

The threat of a human influenza pandemic has greatly increased over the past several years with the emergence of highly virulent avian influenza viruses, notably subtype H5N1 (*9*), and the more recent emergence of subtype H1N1. Federal agencies have modeled the high probability of a serious pandemic influenza outbreak and have begun to institute national and state plans to reduce transmission and mitigate the disease (*10*). The inadequate supply of some vaccines and antiviral medications and insufficient community mitigation planning have led to concern that the United States is inadequately prepared to deal with a pandemic (*11*).

Improving pandemic preparedness is critical, given the catastrophic consequences of influenza pandemics that have occurred in the past century, in 1918, 1957, and 1968; the severity of all past pandemics was substantial, ranging from 700,000 deaths (in 1968) to >50 million deaths (in 1918) (*1*,*11*). Evidence to determine the most effective nonpharmaceutical intervention strategies is limited (*12*). Some strategies being suggested include targeted, layered containment (*13*), which involves antiviral drug treatment for identified case-patients and prophylaxis for and quarantine of their household members, school closures, and social distancing in the community and workplace (*2*,*8*,*13*,*14*). Research has suggested that US adults seem to possess a broad willingness to comply with response strategies that include social distancing, although some segments of the population will likely be less able to comply with isolation recommendations (*8*), particularly those related to isolation from the workplace.

This study may provide public health authorities with realistic expectations for the success or failure of proposed mitigation measures, given that some population subgroups may have less ability to comply with recommendations because of real or perceived job insecurity and financial problems associated with missing work. Our findings suggest that some employment characteristics (inability to work from home, lack of paid sick leave) are associated with working adults’ ability to comply with recommendations and will be major workplace intervention points (areas to target) in the event of a serious outbreak. In addition, sociodemographic characteristics (particularly low-income status) put some workers at disproportionate risk of contracting and spreading pandemic influenza because of their perceived inability to miss work. These assessments may help identify the conditions under which some groups will be disproportionately likely to fail to comply and may help with workplace efforts to plan accordingly and communicate effectively in the event of a serious outbreak of pandemic influenza.

Job insecurity, whether real or perceived, is a real consideration for many working adults. US health authorities recommend that to prepare for a pandemic, businesses should establish policies for nonpunitive liberal leave and flexible worksite accommodations (*2*). However, we know of no legal precedent for mandatory job protection in the event of public health emergencies. Our study found that employees without paid sick leave, those with low income, and those who live in urban areas fear losing their jobs should they comply with recommendations to stay home in the event of a serious outbreak of pandemic influenza. Those respondents who said that they would not be paid if kept from work were almost 5× more likely as those who would receive pay to say that they would lose their job or business as a result of having to stay home from work. We were not surprised by this finding, given the long history of social epidemiology literature (e.g., the Whitehall studies) that has documented the effect of occupational status or grade, organizational injustice, job stress, and workplace power differentials on both job insecurity and disease outcomes (*15*–*20*). The effect of lack of paid sick leave provides insight into a measure of inequality in the work force, such that some groups of employees (e.g., those in minimum wage jobs or without paid sick leave), because of concerns about job security that stem from their workplace status, lack the power to choose to stay home from work in the event of an outbreak. Notably, across all income categories, low- and middle-income workers were significantly more likely than high-income workers to say that they would be likely to lose their job or business as a result of staying home for 7–10 days in the event of an outbreak. Those respondents living in urban areas also were 60% more likely than those living in rural areas to fear job insecurity. This fear could pose substantial problems for pandemic influenza mitigation because those in urban areas may be strongly encouraged to remain isolated to avoid virus spread in conditions of population density and crowding.

Financial problems also are likely to weigh heavily on the minds of US workers during a pandemic, and these problems may be part of the complexity of factors that comprise compliance considerations. Although US health authorities have recommended that businesses develop policies for employee compensation in the event of an influenza pandemic that causes workplace absences (*2*), we know of no precedent requiring that paid sick leave be granted (by employers or state or federal government) to employees who comply with isolation recommendations and miss work in the event of a public health emergency. Our study has elucidated some employment characteristics that are associated with the likelihood that workers think they would experience serious financial problems if they had to miss work; inability to work from home and lack of paid sick leave were associated with reports of experiencing serious financial problems if isolated from work over the 3 periods: 7–10 days, 1 month, and 3 months. Respondent income was another significant predictor of serious financial problems that may limit ability to comply with isolation recommendations. Even relatively short periods of isolation from the workplace (7–10 days) would be a problem for low-income workers, and if an outbreak were serious enough to warrant 1-month isolation recommendations, persons in low- and middle-income groups would have more difficulty complying than would upper-income groups, thus limiting the effectiveness of mitigation strategies. Moreover, at 3 months’ of isolation, persons from all income levels, especially low-income, would likely experience serious financial problems.

Strengths of our study include its practical significance; our findings may help preparedness planners find work-specific strategies that may increase the likelihood of compliance with isolation recommendations. These strategies may include working with employers to ensure work-from-home or sick leave capabilities for nonessential employees and planning to provide state or federal supplementary income support and job protection for workers who would not be paid if they missed work because of official pandemic mitigation recommendations. Other countries have implemented similar measures in emergencies; for example, during the 2003 pandemic of severe acute respiratory disease, the government of Singapore provided financial support to citizens who had to stay home to prevent the spread of the disease (*21*). In the United States, no such measures have been taken to prevent the spread of infectious diseases, but for other emergencies such as hurricanes and floods, federal income support has been provided to victims by way of disaster relief funds. In the event of a serious outbreak of pandemic influenza, when timely action will be needed to encourage and ensure isolation compliance, an existing mechanism for delivering financial support to affected persons is unemployment insurance. Currently, eligibility requirements for this benefit are limited to employees who involuntarily lose their job, but this requirement could be changed to use an existing system to disperse lump-sum payments to those financially affected by a pandemic, if the severity of an outbreak warranted isolation from the workplace for long periods.

Findings from our study should be considered in light of a few limitations, including the 36% response rate. Low response rates can bias samples, reflecting systematic differences between responders and the population from which they were drawn, thus limiting the external validity of estimates (extrapolation to the general population). However, the 1-month period of the survey (and thus limited time for callbacks) may mirror what might be necessary in the event of a pandemic, in which public surveys with a rapid turnaround time are necessary to gauge public knowledge and resource needs in an emergency situation. We point to research that suggests that the results of weighted data from surveys of shorter duration are similar to those based on surveys of longer duration and higher response rates and can be used without an unacceptable risk for bias (*22*,*23*). Furthermore, the HSPH Pandemic Influenza Survey, as it relates to our findings about job insecurity, did not assess perceptions of job loss versus reality of job loss, nor did it assess reasons why some respondents perceived that job loss would be a consequence for missing work for 7–10 days in the event of a serious outbreak. Future population surveys could attempt to disentangle these beliefs to inform policy and communication aimed at enabling compliance with workplace isolation strategies to quell the spread of a future pandemic.
